# AF-SENet: Classification of Cancer in Cervical Tissue Pathological Images Based on Fusing Deep Convolution Features

**DOI:** 10.3390/s21010122

**Published:** 2020-12-27

**Authors:** Pan Huang, Xiaoheng Tan, Chen Chen, Xiaoyi Lv, Yongming Li

**Affiliations:** 1 School of Microelectronics and Communication Engineering, Chongqing University, Chongqing 400044, China; txh@cqu.edu.cn (X.T.); yongmingli@cqu.edu.cn (Y.L.); 2 School of Information Science and Engineering, Xinjiang University, Ürümqi 830046, China; chen9518chen@163.com; 3 College of Software, Xinjiang University, Ürümqi 830046, China; xjuwawj01@163.com

**Keywords:** whole-slide images, cervical cancer, deep convolutional features, feature fusion, image features

## Abstract

Cervical cancer is the fourth most common cancer in the world. Whole-slide images (WSIs) are an important standard for the diagnosis of cervical cancer. Missed diagnoses and misdiagnoses often occur due to the high similarity in pathological cervical images, the large number of readings, the long reading time, and the insufficient experience levels of pathologists. Existing models have insufficient feature extraction and representation capabilities, and they suffer from insufficient pathological classification. Therefore, this work first designs an image processing algorithm for data augmentation. Second, the deep convolutional features are extracted by fine-tuning pre-trained deep network models, including ResNet50 v2, DenseNet121, Inception v3, VGGNet19, and Inception-ResNet, and then local binary patterns and a histogram of the oriented gradient to extract traditional image features are used. Third, the features extracted by the fine-tuned models are serially fused according to the feature representation ability parameters and the accuracy of multiple experiments proposed in this paper, and spectral embedding is used for dimension reduction. Finally, the fused features are inputted into the Analysis of Variance-F value-Spectral Embedding Net (AF-SENet) for classification. There are four different pathological images of the dataset: normal, low-grade squamous intraepithelial lesion (LSIL), high-grade squamous intraepithelial lesion (HSIL), and cancer. The dataset is divided into a training set (90%) and a test set (10%). The serial fusion effect of the deep features extracted by Resnet50v2 and DenseNet121 (C_5_) is the best, with average classification accuracy reaching 95.33%, which is 1.07% higher than ResNet50 v2 and 1.05% higher than DenseNet121. The recognition ability is significantly improved, especially in LSIL, reaching 90.89%, which is 2.88% higher than ResNet50 v2 and 2.1% higher than DenseNet121. Thus, this method significantly improves the accuracy and generalization ability of pathological cervical WSI recognition by fusing deep features.

## 1. Introduction

### 1.1. Background

In recent years, the field of medical image diagnosis has been trending in the direction of artificial intelligence, which has effectively improved the diagnostic efficiency and accuracy of pathologists and reduced missed detections and misdiagnoses caused by human fatigue and insufficient clinical experience [1]. Cancer has always been a major problem among human beings, especially cervical cancer, which has high incidence and mortality rates worldwide. In developed countries, the incidence of cervical cancer is low due to the high level of medical treatment. For example, in the past 30 years, due to advancements in screening and prevention technology in the United States, the incidence of cervical cancer has dropped by approximately 50% [2,3].

Early screening for cervical cancer is an important step in its prevention. Medical screening techniques for cervical cancer include cervical scraping, human papillomavirus (HPV) examination, colposcopy, HPV–ThinPrep cytologic test combined detection, naked-eye observation, and liquid-based cell detection [4,5,6,7].

After detecting cervical lesions through the abovementioned early screening methods, a follow-up biopsy is required for pathological diagnosis. Pathologists use circular electric cutters to perform the conization of cervical lesions and obtain tissue sections. Alternatively, they use biopsy forceps to obtain tissue from lesions, make full slides and use them to generate whole-slide images (WSIs) by microscopic imaging, and then make glass slides for microscopic examination. This is the established standard for cervical cancer diagnosis. Recently, it has become common to create WSIs of the slides for use in cancer diagnosis [8]. Therefore, the key issue in cervical cancer diagnosis has become how to identify WSIs quickly and accurately during different periods. Currently, pathologists classify cervical biopsy images according to the “WHO Classification of Tumors of Female Reproductive Organs, Fourth Edition”, mainly by analyzing the number of atypical cells in the biopsy image, the speed of cell mitosis, the level of cell differentiation, the number of atypical cells in the nucleus, the presence of polarity disorders, and the degree of surface cell keratinization [9]. However, biopsy images of healthy people and images of low-grade squamous intraepithelial lesions (LSILs) are highly similar, and the classification task is difficult and depends largely on the experience of the pathologists. Therefore, the early identification of cervical lesions in images is a significant challenge for medical institutions with pathologists who have only a few years of experience or no professional pathologists at all [10].

### 1.2. Related Work

In image processing, due to the wide variety of images and the large number of types of images, the color, texture, shape, and spatial relationship features of a single image are highly complex, and describing these image features has become a key problem. The extraction of traditional image features often requires researchers to have rich and solid professional knowledge. In the development and adjustment stage of an algorithm, designing features manually occupies much time and energy for the researchers, and the final results often remain dependent on experience and luck to a certain extent. With the development of neural networks, deep convolutional networks have provided researchers with new ideas of image feature extraction and sample representation. The sample can be automatically transformed from a high-level abstract representation to a series of linear combinations and nonlinear transformations stacked layer by layer. By extracting the feature information of an image, one can effectively avoid the inefficiency and cumbersomeness of manually designing features [11,12,13,14].

Furthermore, deep networks of multiple hidden layers are often able to provide a more profound and essential characterization of the original input data than networks with a single hidden layer, thereby learning more advanced data feature representations [15,16].

For pathological images of breast cancer, brain cancer, and lung cancer, there have been many examples of using deep convolutional network models for feature extraction and sample representation, and good results have been achieved [17,18,19,20,21,22,23,24].

Scientific research teams across the world use cervical WSIs to diagnose the degree of cervical cancer and the accuracy of WSI classification is relatively high when the degree of the disease varies greatly, especially when the classification accuracy of normal and cancerous images is almost 100%, but the overall classification accuracy is low. P. Huang et al. [25] proposed a method for the classification of pathological cervical images based on the least absolute shrinkage and selection operator (LASSO) and ensemble learning-support vector machine, and they explored the classification relationship between images of different stages in a comprehensive manner; for pathological tissue images with large differences in disease degrees, the upper classification effect was good, especially for the normal and cancerous images, which reached 99.24%, but the recognition accuracy of early lesions was only 84.25%, and the average classification accuracy was not high. Wang, YH et al. [26] proposed a computer-assisted diagnosis system for cervical intraepithelial carcinogenesis using ultra-large-scale cervical histology images to diagnose cervical intraepithelial neoplasia (CIN) along the vertical axis of the squamous epithelium. The changes in nuclear structure and morphology were quantified and classified, and the multiclass support vector machine (SVM) was used to classify 31 case images with an accuracy of 94.25%, but the classification objects were not sufficiently comprehensive, and the clinical practicality was not high. This system has a certain practical value in assisting scientists with case diagnosis and doctor training. This article focuses only on CIN image classification, and there is insufficient research on healthy tissue images and tissue images in the early stages of diseases. Wei L. et al. [27] proposed an automatic detection method of cervical cancer that analyzes and extracts texture features via the gray-level co-occurrence matrix (GLCM) of case images and uses the k-means and marker-controlled watershed algorithms to segment and fuse the images. The GLCM and pathological area characteristics are obtained, and finally, SVM is used for classification and recognition. The classification accuracy of the recognized cancer under normal classifications is 90%, but the early classification accuracy of lesion tissue images is only 70%. The classification objects are not sufficiently comprehensive, and the average classification accuracy rate is not high enough to achieve a strong clinical diagnosis effect. Guo P. et al. [28] proposed an automated and localized method based on fusion to assess abnormalities in cervical cancer tissues. After using SVM and the linear discriminant analysis method by a vote on the vertical stages of 61 image cases, the highest accuracy rate of CIN classification was 88.5%, and the overall classification effect was not good, especially in the recognition of healthy people’s cervical tissue and tissue in the early stages of diseases. Keenan S.J. et al. [29] developed a machine vision system using the KS400 macro programming language and tried to use automatic machine vision to develop an objective scoring system for 230 CIN images at all levels of disease. The classification effect for normal and CIN3 images in the scoring system was as high as 98.7%, and the accuracy rate for microcytosis and CIN1 images was 76.5%. This article did not explore the possible relationship between cervical cancer stages and CIN or between normal and microcytosis tissue biopsy images.

However, for the classification of pathological cervical cancer tissue images, in the method to extract shallow convolutional networks and traditional image features, due to high redundancy, low correlation, low sparseness, and sample indication problems, such as weak classification ability, the recognition result is poor [10].

### 1.3. Aims of the Study

(1) This research aims to solve the problems of missed detection and misdiagnosis caused by the high similarity of cervical pathological tissue images and reliance on the experience of pathologists and to solve the problem of low overall screening efficiency caused by large reading data.
(2) The purpose of this research is to explore further the influence of fusion depth features of the four classification effects of tissue images of the new classification standard for cervical cancer tissue images.

## 2. Method

### 2.1. Dataset

**Figure 1:**
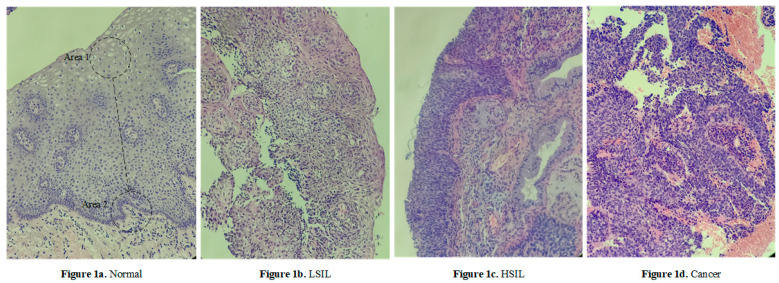
Cervical pathological tissue image, each with a resolution of 3456 × 4608 (RGB).

**Figure 2:**
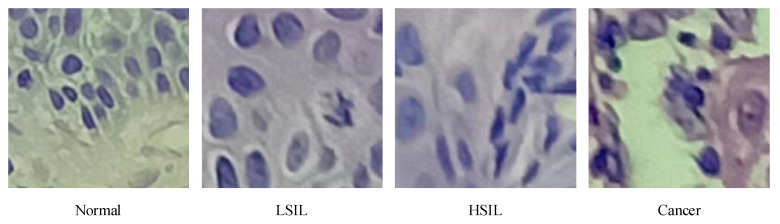
Small-size cervical tissue biopsy image, each with a resolution of 200 × 200 (RGB) pixels.

The cervical tissue biopsy image dataset used in this article came from the First Affiliated Hospital of Xinjiang Medical University. These data were reviewed by the Medical Ethics Committee and were desensitized. The patients’ permission was obtained. There were 468 RGB images in total, each with a resolution of 3456 × 4608, as shown in Figure 1, of which 150 were normal, 85 were low-grade squamous intraepithelial lesion (LSIL), and 104 were high-grade squamous intraepithelial lesion (HSIL). There were 129 images of cancer. After processing via the image enhancement method proposed in this article, the enhanced, small-size cervical tissue biopsy image dataset had a total of 100,020 images, each with a resolution of 200 × 200 (RGB) pixels, as shown in Figure 2, including 50,370 normal images, 11,914 LSIL images, 16,677 HSIL images, and 21,059 cancer images. Among them, 90% of the images were used as the training set, and 10% were used as the test set, as shown in Figure 2.

In Figure 1, it was observed that the epithelial cells showed an increase in the number of atypical immature cells from the top (Area 1) to bottom (Area 2). In addition, as the degree of lesions increased, the number of atypical immature cells in the cervical biopsy tissue images also increased. This number of cells increased sequentially and became increasingly cancerous, which was reflected in the phenomenon that the nucleus-to-cell ratio of the cells became larger, and the cytoplasm deepened and became thicker. We found that the difference between Figure 1a,b was small, and the difference between Figure 1c,d was small. The cell morphology in the four pictures was varied and contained very rich information, and the similarity was very high. From this perspective, it is very difficult to describe the cervical pathological tissue image comprehensively through the use of traditional image features, which leads to an unsatisfactory final classification effect, especially in the early stage of disease.

#### 2.1.1. New Classification Standards

The naming scheme of the WHO (2014) classification of female reproductive system tumors was used for cervical squamous cell precancerous lesions (Table 1), where LSIL is defined as a kind of clinical and pathological change caused by HPV infection. Squamous intraepithelial lesions have a low risk of canceration currently or in the future. Synonyms for LSIL include cervical intraepithelial neoplasia Grade I (CIN1), mild atypical hyperplasia, flat condyloma, and keratocytosis. HSIL is defined as follows: If left untreated, this squamous intraepithelial lesion has a significant risk of progressing to invasive cancer. Synonyms for HSIL include cervical intraepithelial neoplasia grade II (CIN2), cervical intraepithelial neoplasia grade III (CIN3), moderate atypical hyperplasia, severe atypical hyperplasia, and squamous epithelial carcinoma in situ [2].

#### 2.1.2. Introduction to Image Features

**Figure 3:**
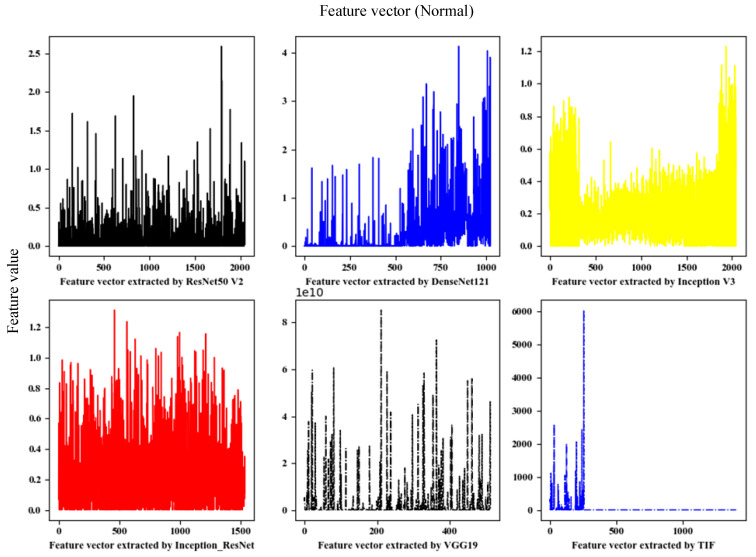
Feature vector extracted by deep model and Traditional image features (TIF).

This paper used the fine-tuned deep network model to extract deep convolution features. A total of five depth models were trained. The dimensions of the extracted deep convolution features are shown in Table 2, and the feature visualization is shown in Figure 3.

**Traditional image features (TIF):** This paper mainly used a local binary pattern (LBP) and a histogram of oriented gradient (HOG) to extract features separately and then serially merged them into TIF vectors.

**Figure 4:**
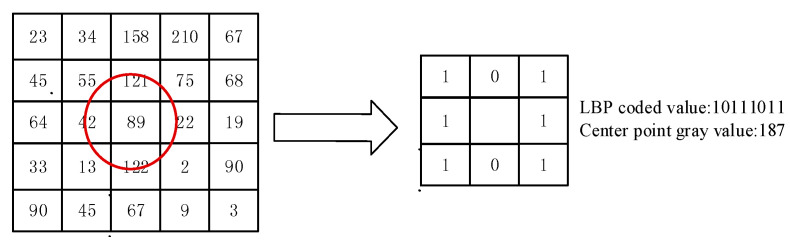
Diagram of the local binary pattern (LBP) descriptor calculation process.

This paper used an LBP [30,31] algorithm to extract image texture features. LBP is a parameterless texture descriptor. LBP has the advantages of being simple and effective and having a strong recognition ability and low computational complexity. The gray value extracted by LBP was used to draw the gray statistics histogram, and the specific method is shown in Formula (1). A neighborhood in standard LBP is defined by a radius because square neighborhoods do not cover the entire image. The gray values of each circular neighborhood were obtained by comparing the gray values of the pixels on the circular border with the center pixel and then clockwise encoding at 90 degrees to obtain a re-encoded grayscale image in turn. The specific process is shown in Figure 4. The form of the LBP descriptor is shown in Formula (2).

(1) $$H_{f}\left(k\right)=\frac{n_{k}}{n},n=1,2,\ldots ,M-1$$

where $H_{f}\left(k\right)$ represents the frequency of gray value k after encoding, *n* represents the number of pixels in the numbered image, $n_{k}$ represents the sum of pixels of the gray value k, and M represents the number of gray values in the encoded image.

(2) $$LBP_{R,N}\left(g_{o}\right)=\sum_{i=0}^{N-1}p\left(g_{i}-g_{o}\right)\times 2^{i},p\left(x\right)=\{\begin{array}{c} 1,x\leq 0\\ 0,x>0 \end{array}\;,\;R=\sqrt{{\left(x_{1}-x_{2}\right)}^{2}+{\left(y_{1}-y_{2}\right)}^{2}}$$

where R represents the radius of the circular neighborhood. The minimum unit is the Euclidean distance D between the four neighboring pixels of the image, and the distance is 1. Calculated through the defined R formula, the D value of the center pixel four-neighborhood is 1, the D value of the eight-neighborhood is 2, the D value of the 16-neighborhood is 3, and so on. *n* represents the number of pixels in the circular area, and $g_{o}$ and $g_{i}$ represent the gray values of the central pixel and the i-th pixel in the circular neighborhood, respectively. When R = 1, the boundary point is the eight-neighborhood of the center pixel, and when R = 2, *p* = 16, the center pixel and the eight-neighborhood are considered as a whole to form a new center pixel. The boundary point is the 16-neighborhood of the new central pixel, and so on.

HOG [32,33] features have a strong image structure and contour description capabilities as well as a strong recognition effect on the description of local areas. HOG features are also suitable for describing texture features. Texture features have local irregularity and macro regularity. Using appropriate HOG cell units to divide the image and extracting HOG features can obtain the changing pattern of the overall texture features of the image. Choosing HOG cell units that are too small results in local features that are too fine and macro features that are unclear and computationally complex. If the selected HOG cell unit is too large, the local feature description is incomplete, which is not conducive to generalizing the macro features. The cell unit size used in this paper was 10 × 10.

### 2.2. Image Processing

**Random cropping based on grayscale matching:** Random cropping was performed for each cervical image. The cropped size was 200 × 200 × 3, but there were images without cell nuclei. Obviously, such an image was useless at the training of the depth model. The sum of absolute differences (SAD) [34] used grayscale matching to remove such cropped subimages, as expressed in Formula (3) and manually cropped the verification set to obtain the optimal threshold $D_{T}$. The random cropping function is defined as $y_{p}^{m}=Crop\left(y_{s}^{n},size\right)$, and its core formula is shown in (4) and (5).

(3) $$D\left(i,j\right)=\sum_{s=1}^{M}\sum_{t=1}^{N}\left|S\left(i+s-1,j+t-1\right)-T\left(s,t\right)\right|$$

(4) $$y_{p}^{m}=y_{s}^{n}\left(i*d_{1}+p:\left(i+1\right)*d_{1},j*d_{2}+q:\left(j+1\right)*d_{2},z\right)$$

(5) $$\{\begin{array}{c} d_{1}=size\left[0\right]\\ d_{2}=size\left[1\right]\\ z=z \end{array}\;,\{\begin{array}{c} 0\leq i\leq x/size\left[0\right]\\ 0\leq j\leq y/size\left[1\right] \end{array}\;,\{\begin{array}{c} p=rand\left(k\right),0\leq k\leq x-d_{1}\\ q=rand\left(w\right),0\leq w\leq y-d_{2} \end{array}$$

where $d_{1}$ represents the length of the row of the image matrix; $d_{2}$ is the length of the column, i, j represent the variables of the number of cropping times; x, y represent the lengths of the row and column of the original image matrix, respectively; k, w represent the value range of randomly cropped row and column position variables; p,q represent randomly generated row and column values; S represents the cropped image matrix; T represents the template cropped image that meets the requirements; and s,t are used to match the row and column variables of the cropped image.

**Random translation:** The random translation function is defined as $y_{p}^{k+1}=Warp\left(y_{p}^{m}\right)$. When implementing this function, a movement matrix M is constructed first. The specific formula is shown in (6).

(6) $$y_{p}^{k+1}=y_{p}^{m}*M\;,\{\begin{array}{c} M=\left|\begin{array}{ccc} 1 & 0 & t_{x}\\ 0 & 1 & t_{y} \end{array}\right|\\ t_{x}=rand\left(x\right),t_{x}=rand\left(y\right) \end{array}$$

**Random rotation:** The random rotation function is defined as $y_{p}^{k+2}=Rotation\left(y_{p}^{m}\right)$. When implementing this function, a rotation matrix M is constructed first. The specific formula is shown in (7).

(7) $$y_{p}^{k+2}=y_{p}^{m}*M\;,\{\begin{array}{c} M=\left|\begin{array}{cc} cos\theta & -sin\theta \\ sin\theta & cos\theta \end{array}\right|\\ \theta =rand\left(360\right) \end{array}$$

**Random zoom:** The random zoom function is defined as $y_{p}^{k+1}=Zoom\left(y_{p}^{m}\right)$. Zooming is performed by dividing the image and selection points. The specific formula is shown in (8).

$$y_{p}^{k+3}=y_{p}^{m}\;\left(0:m:x,0:n:y,z\right),$$

(8) $$\{\begin{array}{c} m=rand\left(size\left[0\right]\right)\\ n=rand\left(size\left[1\right]\right) \end{array}$$

**Random brightness adjustment:** The random brightness adjustment function is defined as $y_{p}^{k+4}=Bright\left(y_{p}^{m}\right)$. When implementing this function, a movement matrix M is constructed first. The specific formula is shown in (9).

$$y_{p}^{k+4}=y_{p}^{m}\;\left(x,y,z+k\right),$$

(9) k=−rand(255)+rand(255)

**Image normalization:** To prevent the information on the low-value area from being concealed by the information on the high-value area, the image is normalized.

(10) $$Y_{o}=Y_{o}/255.0$$

The pseudocode for the image enhancement process of this article is shown in **Algorithm 1**.

**Algorithm 1** Image Enhancement Processing **Input:**
$Y_{I}$. **Output:**
$Y_{o}$1  $Y_{I}$ is the original image matrix, $Y_{o}$ is the enhanced image matrix.2  **FOR**
*p* = 1: *s* //*s* is the number of image samples3    Implement random cropping based on grayscale matching for $y_{s}^{n}$:4    (i) Perform random cropping, according to Formulas (4) and (5):5      $y_{p}^{m}=Crop(y_{s}^{n},size)$.6    (ii) Determine whether the following conditions are met: $D\left(i,j\right)<D_{T}$7      $Y_{o}=Y_{o}\cup y_{p}^{m}$8 Randomly shift the randomly cropped image tensor $y_{p}^{m}$ according to Equation (6):9    $y_{p}^{k+1}=Warp\left(y_{p}^{m}\right)\;then\;Y_{o}=Y_{o}\cup y_{p}^{k+1}$10  Randomly rotate $y_{p}^{m}$ according to Formula (7):11    $y_{p}^{k+2}=Rotation\left(y_{p}^{m}\right)\;then\;Y_{o}=Y_{o}\cup y_{p}^{k+2}$12   Randomly scale $y_{p}^{m}$ according to Formula (8):13    $y_{p}^{k+3}=Zoom\left(y_{p}^{m}\right)\;then\;Y_{o}=Y_{o}\cup y_{p}^{k+3}$14   Randomly adjust the brightness of $y_{p}^{m}$ according to Equation (9):15    $y_{p}^{k+4}=Bright\left(y_{p}^{m}\right)\;then\;Y_{o}=Y_{o}\cup y_{p}^{k+4}$16  Normalize the enhanced, small-sized image according to Equation (10):17    $Y_{o}=Y_{o}/255.0$18 **END**19 **Return**
$Y_{o}$

### 2.3. Fine-Tuned Transfer Model

In this paper, the Calling the Applications module of the deep learning framework Keras, and the DenseNet121, ResNet50 v2, Inception v3, and Inception-ResNet models were pre-trained on ImageNet [35] data and used for transfer learning. Because the images in ImageNet had a large gap between images of cervical pathology, and the image features recognized by the top convolutional layer were more abstract and specific, this paper used only the weight of the 1st convolutional layer of the pre-trained model.

The last layer of the deep network model pre-trained on ImageNet was excessively specialized, and the last layer (pre-trained models of applications of Keras) was obviously not suitable for transfer learning. Thus, this layer was deleted.

The most important aspects of transfer learning are the setting of the learning rate, the selection of the loss function, the configuration of the optimizer, and the measures for the prevention of overfitting.

The loss function is the objective function in transfer learning, and it is an indicator of the directions of weight changes. The choice of the loss function directly determines the quality of the result of transfer learning. This article used the categorical cross-entropy (CE) [36] function as the loss function. The basic principle is shown in Equation (11):

(11) $$CE\left(x\right)=-\sum_{i=1}^{C}y_{i}logf_{i}\left(x\right)$$

where x represents the input sample, C is the expected total number of classifications, $y_{i}$ is the i-th true label, and $f_{i}\left(x\right)$ corresponds to the output value of the model.

The optimizer is also one of the most important parameters in transfer learning. In this paper, stochastic gradient descent (SGD) [37] was used as the optimization algorithm. It updates only once per epoch without redundancy and is fast. The basic principle is shown in Equation (12).

(12) $$\theta =\theta -\eta \cdot \nabla_{\theta }J\left(\theta j;x^{\left(i\right)};y^{\left(i\right)}\right)$$

where η is the learning rate, also known as the step size, which is one of the most important parameters in transfer training. An excessively large learning rate causes the gradient to disappear, so the optimal solution cannot be found or the convergence time is too long. The learning rate was 0.1 for epochs 0–60, 0.01 for epochs 61–120, 0.001 for epochs 121–180 epochs, and 0.0001 for epochs 181 and above in this paper. $x^{\left(i\right)}$ represents the sample data onto the i-th epoch.

**Figure 5:**
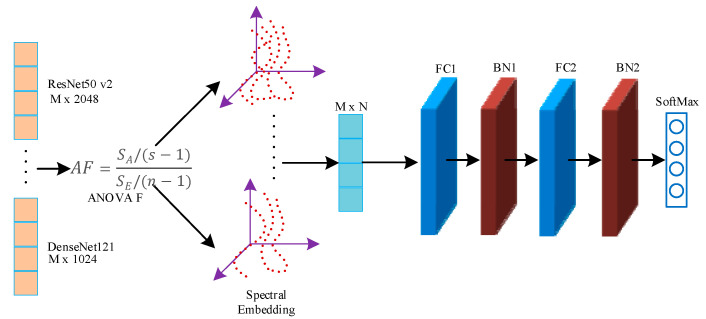
Framework diagram of the deep network convolutional feature fusion subnet based on ANOVA F-spectral embedding. The loss function of this subnet was the categorical cross-entropy function, the optimization algorithm was stochastic gradient descent, and the learning rate was: 0.1 for epochs 0–60, 0.01 for epochs 61–120, 0.001 for epochs 121–180, and 0.0001 for epochs 181 and above. In the Figure 5, M represents the number of samples in the training set, and *n* represents the feature-length of each sample after dimension reduction and fusion. FC stands for Full Connection layer, BN stands for batch normalization layer, and s is the length of the column in the depth feature matrix (the feature length of the sample, n is the length of the sample.

In transfer learning, overfitting occurs frequently and has a large impact on the training results. The main method to prevent overfitting is data augmentation. In addition, in the top layer designed in this paper, the convolution kernel was regularized, and the dropout layer and batch normalization [38] layer were added after the full connection in Figure 5. The relevant parameters of the fully connected layer and the regularization layer are shown in Table 3.

The regularization and processing algorithm used in this paper was LASSO [39], and the basic principle implemented in the convolution kernel is shown in Equation (13).

(13) $$min_{w}\sum_{i=1}^{m}{\left(y_{i}-w^{T}x_{i}\right)}^{2}+\lambda ∥w{∥}_{1}$$

where $y_{i}$ represents the predicted label value, $w^{T}x_{i}$ represents the predicted label value, λ represents the L1 regularization coefficient, and $\lambda ∥w{∥}_{1}$ represents the L1 regularization processing on the weight.

The forward calculation formulas for the dropout layer [40] used in the article are shown in Equations (14) and (15):

(14) $$r_{j}^{\left(l\right)}\sim Bernoulli\left(p\right)\;,y_{avr}^{\left(l\right)}=r^{\left(l\right)}*y^{\left(l\right)}$$

(15) $$z_{i}^{\left(l+1\right)}=w_{i}^{\left(l+1\right)}y_{avr}^{\left(l\right)}+b_{i}^{\left(l+1\right)}\;,y_{i}^{\left(l+1\right)}=f\left(z_{i}^{\left(l+1\right)}\right)$$

where $r_{j}^{\left(l\right)}$ obeys the Bernoulli binomial distribution of probability p and $r^{\left(l\right)}$ is the generated 0,1 vector. By setting the activation value of 0, some nodes in layer l of the network stop working, and for the input of layer l+1, only the nonzero nodes in layer l+1 are considered (in fact, all nodes are considered, but the output is a node of 0 that has no effect on the next layer of the network, and it cannot update its related network weights during backpropagation).

### 2.4. Deep Convolution Feature Fusion Mechanism Based on Analysis of Variance-F Value-Spectral Embedding (AF-SENet)

In this paper, the DenseNet121, ResNet50 v2, Inception v3, and Inception-ResNet models were pre-trained on the ImageNet dataset and then transferred to the pathological cervical tissue image dataset for further fine-tuning [41]. The different trained models may contain complementary information. To explore this possible information complementarity, this paper proposed the use of the analysis of variance-F value (ANOVA F)-spectral embedding strategy to analyze the changes in the ANOVA F values for different fusion combinations. Spectral embedding [42] was then used for fusion mapping. The softmax classifier was used for classification. The fused subnet is own in Figure 5.

In this section, the deep convolutional network feature tensor extracted from a single model after migration fine-tuning is represented by $X=\left\{X_{1}^{s},\cdots X_{n-1}^{s},X_{n}^{s}\right\}$, where n represents the number of samples and s is the length of the row in the depth feature matrix (the feature-length of the sample). In this section, ANOVA F was used to evaluate the redundancy and correlation between different combinations of deep convolutional features. Analysis of variance (ANOVA) mainly explores the contributions to features of the between-group variance and within-group variance in datasets. The definition of the variance value is shown in Equations (16) and (17).

(16) $$group\;error:\;S_{A}=-\sum_{i=1}^{n}n_{i}{\left(\bar{X_{i}}-\bar{X}\right)}^{2}$$

(17) $$Within-group\;error:\;S_{E}=-\sum_{i=1}^{s}\sum_{j=1}^{n}n_{i}{\left(\bar{X_{j}^{i}}-\bar{X_{i}}\right)}^{2}$$

According to Formulas (16) and (17), the test statistic f can be constructed as follows:

(18) $$f=\frac{S_{A}/\left(s-1\right)}{S_{E}/\left(n-1\right)}$$

where $S_{A}$ represents the sum of variance values between different samples in the depth feature sample matrix and $S_{E}$ represents the sum of variance values between different features in the depth feature sample matrix.

This paper proposed the ANOVA F-spectral embedding algorithm to select sample features and reduce the dimension to reduce the time complexity of training the subnets under the premise of ensuring high classification accuracy. First, a selection was performed by using the test statistic f. The f-value of each feature of the sample image feature matrix X was calculated. Second, the f-values of the sample features were summed to obtain the total value defined as sum_f. The average f-value ($\bar{f}$) was constructed to measure the importance of each feature of the entire feature set; $\bar{f}$ is shown in Equation (19).

(19) $$\bar{f}=\frac{f_{i}}{\sum_{i}^{n}f_{i}}$$

According to the size of $\bar{f}$, the features were sorted in descending order, and the $sum\_\bar{f}$ of the first i sample features was calculated. If $sum\_\bar{f}$ > 99.9%, the feature selection process was stopped, and the subsequent features were eliminated.

Based on the above selection of features, there were many redundant features. The effect of traditional feature selection methods of objective functions (labels) is not ideal, and linear methods, such as Principal Components Analysis (PCA) and Linear Discriminant Analysis (LDA), can be easily used to perform feature space transformations. The loss of the nonlinear relationships to samples is meant to avoid these problems. The ANOVA F-spectral embedding algorithm is shown in **Algorithm 2**.

**Algorithm 2** ANOVA F-spectral Embedding **Input:**
$X_{I}$.  **Output:**
$X_{o}$ 1   $X_{I}$ is the sample image feature matrix, $X_{o}$ is the selected and transformed sample image feature matrix. 2   **FOR**
*i* = 1: *n* //*n* is the dimension of a feature in the feature matrix of the image sample. 3   Calculate the f-value of each feature according to Formula (18). 4   $f_{vector}=f_{vector}\;U\;f$ 5   $sum_{f}+=f_{i}$//$sum_{f}$ is the sum of the f-values of the sample features, $f_{i}$ is the f-value of the i-th feature. 6  **END** 7  Calculate the ${\bar{f}}_{vector}$ value of each feature according to Formula (18), sort ${\bar{f}}_{vector}$ in descending order 8 **FOR**
*i* = 1: *n* 9  **IF** (sum + = ${\bar{f}}_{vector}\left(i\right)$) < 99.9% 10   X = $X_{I}$(i) 11   **END** 12 **END** 13  Transform X into a graph representation using the affinity (adjacency) matrix representation. 14  Construct an unnormalized Laplacian graph as L=D−A and a normalized graph as $L=D^{-1/2}\left(D-A\right)D^{-1/2}$. 15  Perform eigenvalue decomposition on the Laplacian graph after performing the above treatment on $X_{o}$. 16 **Return**
$X_{o}$

### 2.5. Feature Analysis

For the problem of feature classification, there are a variety of indicators to evaluate the pros and cons of features, including the correlation between features and categories, the redundancy of the features themselves, and the sparsity of features in the feature matrix. In this paper, to explore the advantages and disadvantages of deep convolutional network features and traditional image features of cervical cancer as well as the ability to represent image samples, the chi-square test (Chi2) is shown in Equation (20), and the ANOVA F(AF) test is shown in Equations (16)–(18). These tests explore the redundancy and correlation of features, using the average tree attributes of extremely randomized trees (ETs) [43] to measure the importance of each feature.

(20) $$CHI\left(x,y\right)=\chi^{2}\left(x,y\right)=\sum \frac{{\left(A-T\right)}^{2}}{T}$$

where A is the actual value, and T is the predicted value.

(21) $$\bar{x}=\frac{x_{i}}{\sum_{i}^{n}x_{i}}$$

**Figure 6:**
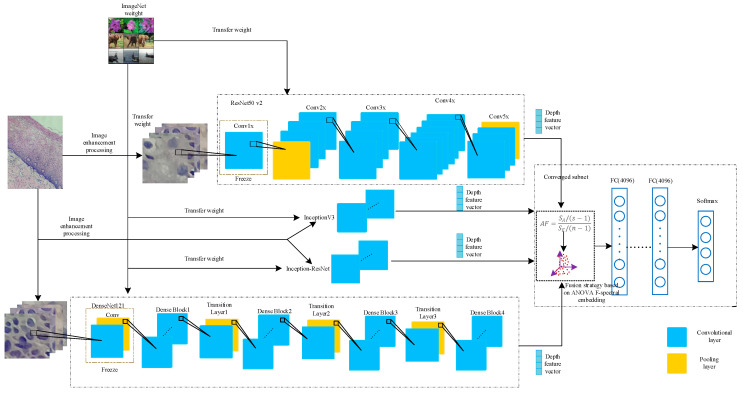
The framework of the proposed method of pathological cervical image classification is based on fusion deep network convolutional features.

Based on the introduction to Section 2.1, Section 2.2, Section 2.3, Section 2.4 and Section 2.5, this article drew the overall implementation in Figure 6.Block diagram of the AF-SENet algorithm, as shown in Figure 6. The complete process includes using the DenseNet121, ResNet50 v2, Inception v3, and Inception-ResNet models pre-trained on ImageNet data and freezing the lowest layers. The pre-training model was used to extract the deep convolution features, and the ANOVA F-spectral embedding algorithm was used for dimension reduction. Serial fusion was performed, and the training subnet was input. The training subnet had two fully connected layers (the number of neurons is 4096) and an output layer, which contained a four-class softmax classifier.

### 2.6. Evaluation Criteria

In this article, the Receiver Operating Characteristic (ROC) curve was used to evaluate the generalization ability of the model. The ROC curve is one of the most commonly used indicators in the evaluation of artificial intelligence models. The true-positive rate (TPR) was calculated, as expressed in Formula (22), and the false-positive rate (FPR) was calculated, as expressed in Formula (23) each time, with the TPR as the vertical axis of the ROC curve and the FPR as the horizontal axis of the ROC curve.

(22) $$TPR=\frac{TP}{TP+FN}$$

(23) $$FPR=\frac{FP}{TN+FP}$$

where TP, FN, FP, and TN are the true-positive examples, false-negative examples, false-positive examples, and true-negative examples, respectively, in the confusion matrix of the classification results.

Suppose that the sample size of the data to be analyzed by ROC is m, and the number of classifications of the sample is *n*, so a label matrix L of [m, n] can be obtained. The value is 0 or 1. Correspondingly, if we predicted the probability that each sample would fall into each category of the outcome of the statistical model, we could also obtain a probability matrix *p* of [m, n], and the value of the matrix *p* was 0–1.

Micro method: Expand the matrices L and *p* by rows, and form two columns of length mxn after transposing. In this way, the multicategory outcome can be converted into a two-category situation, followed by the classic two-category outcome. ROC analysis is sufficient.

## 3. Results and Discussion

### 3.1. Experimental Conditions

All program codes in this article were developed based on the Python language, and the specific software and hardware configurations are shown in Table 4.

### 3.2. Multitype Features and Fusion Analysis

In this paper, the depth features mainly extracted the convolutional features and TIFs of the DenseNet121, ResNet50 v2, Inception v3, Inception-ResNet, and VGG19 models, and the TIFs used the HOG features. LBP features were obtained by serial fusion. To intuitively judge the pros and cons of the indicators through the data distribution, this paper analyzed the Chi2, AF, ETs, and SP of the traditional image features arranged in descending order.

**Figure 7:**
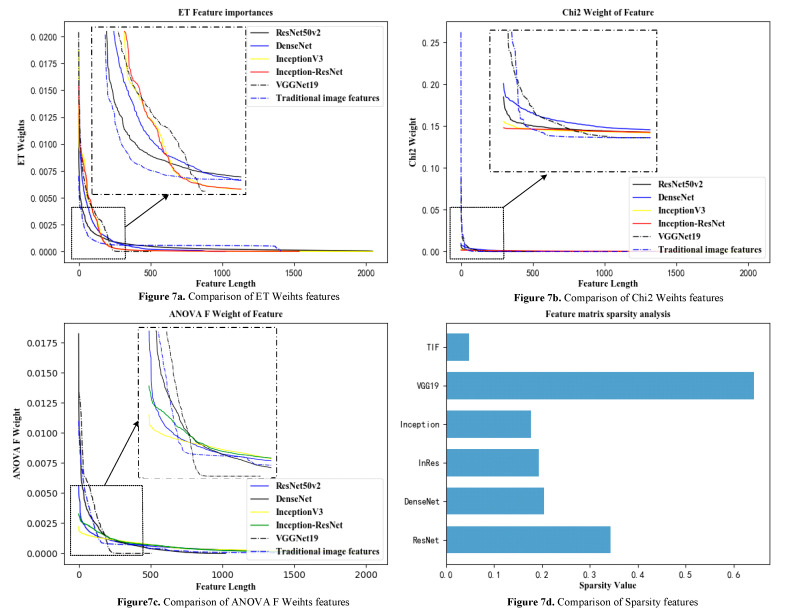
Analysis diagrams for deep convolutional features and traditional features of cervical cancer tissue images. (The method for calculating the weights in this figure is shown in Equation (21).) VGG19 means VGGNet19, InRes means Inception-Resnet, Resnet means ResNet50 V2, DenseNet means DenseNet121, and Inception means Inception V3.

In Figure 7a–c, the abscissa represents only the feature-length, not the specific feature number. To observe the effects on the AF and Chi2 indicators for a single feature of the entire sample, as well as their changes, the weight was normalized, and the calculation formula is shown in Equation (20).

First, this paper combined the fine-tuned DenseNet121, ResNet50 v2, Inception v3, and Inception-ResNet models. The combined relationship and number codes are shown in Table 2.

From the analysis of Table 2 and Figure 7, it can be seen that the sparseness of deep convolutional features was higher than that of traditional features, and the individual contributions to deep convolutional features were more balanced with indicators, such as ET weight, ANOVA F weight, and Chi2 weight, and the indicator curve dropped. The sparseness of the VGG19 model was slightly abnormal.

### 3.3. Accuracy of Classification before and after Fusion

**Figure 8:**
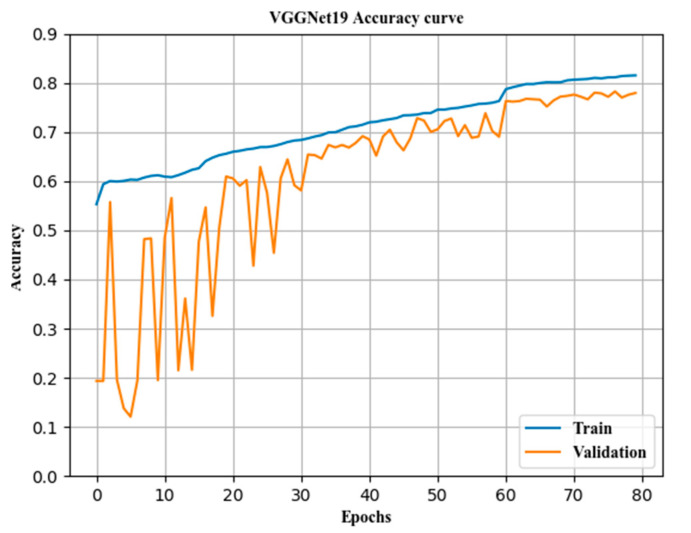
The classification accuracy curve of the VGGNet19 model.

**Figure 9:**
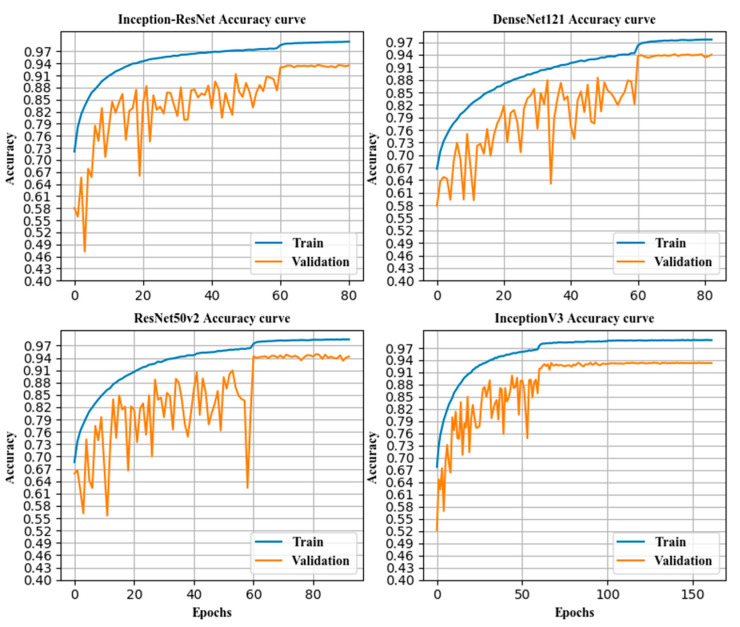
The classification accuracy curve of some individual models.

**Figure 10:**
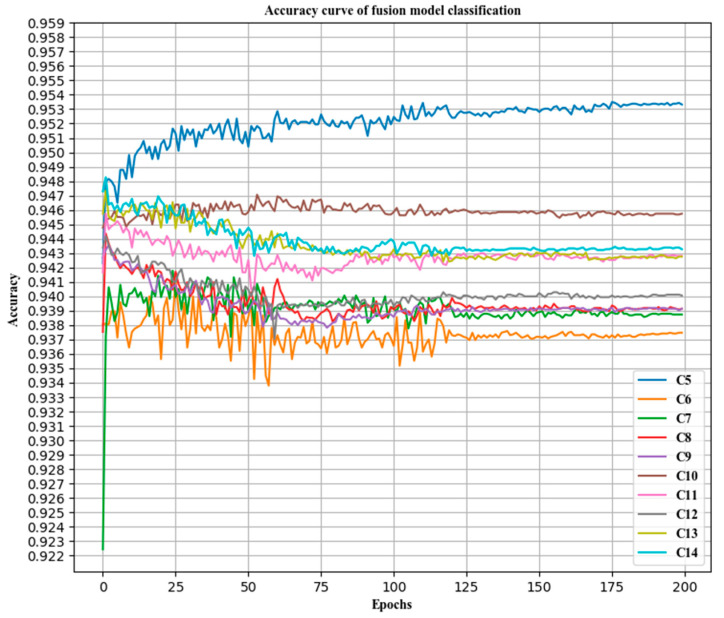
The classification accuracy curves of the fused models.

Various models were compared. The combined classification accuracy rate and the classification accuracy rate of each subcategory were compared with those of a single model, and a horizontal comparison was performed with the VGGNet19 model, as shown in Table 5 and Figure 8, Figure 9 and Figure 10.

### 3.4. Model Evaluation

**Figure 11:**
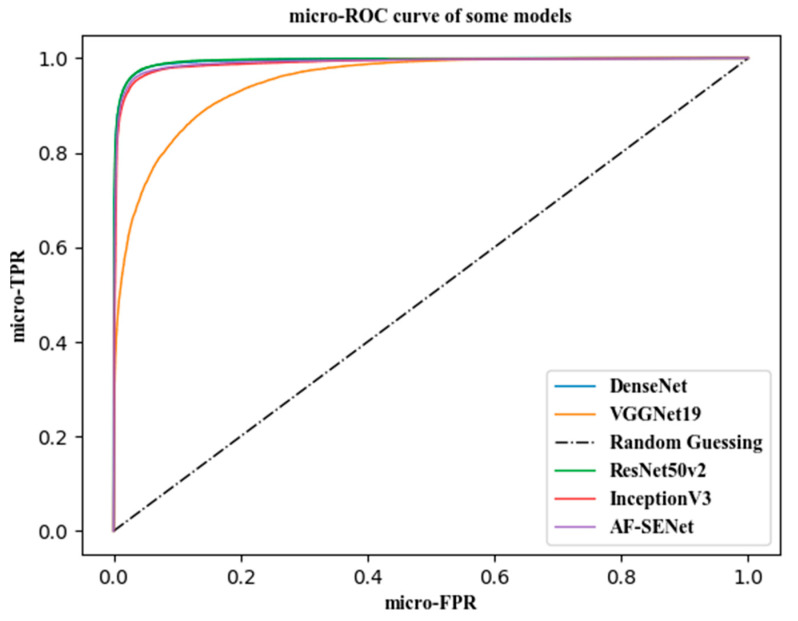
ROC curves of some models.

The ROC curve was used to evaluate the single models, the combined models, and the VGGNet19 model. The evaluation effects are shown in Figure 11, and the Area Under Curve (AUC) value of each model is shown in Table 6.

Since the micro-ROC and micro-AUC values were used as the evaluation indicators for the models, the number of images after data enhancement reached 100,000, and the test sample had more than 17,000 images. The gaps between the ROC curves of the models were not particularly large, most of the curves had only slight differences between them, and the drawing effect was not good. This paper combined the convolutional features of the ResNet50v2 and DenseNet121 models to perform the best classification. The micro-AUC reached 0.9989, and the average classification accuracy rate was 95.29%. The effect of using VGGNet19 was the least ideal. The micro-AUC was only 0.9506, and the average classification accuracy was only 78.37%. Using the traditional image features to classify images by the AF-SENet model proposed in this paper, the micro-AUC was only 0.8952, and the classification accuracy was only 68.31%.

By combining Table 5 and Table 6, we can find that the fused combination of ResNet50 v2-DenseNet121 ($C_{5}$) had an average improvement in classification accuracy of 1.07% over that of ResNet50 v2 and a 1.05% improvement over that of DenseNet121. The recognition ability of the fused model on LSIL was especially improved, reaching 90.89%, which was an increase of 2.88% over ResNet50 v2 and an increase of 2.1% over DenseNet121. For the micro-AUC, the fused model achieved an increase of 0.0018 over ResNet50 v2 and an increase of 0.001 over DenseNet121. The fused combination of Inception v3 and Inception-Resnet ($C_{10}$) had an improvement in average classification accuracy of 1.61% over Inception v3 and an improvement of 0.97% over Inception-Resnet. It also achieved an improvement in micro-AUC of 0.0027 over Inception v3, which was an increase of 0.0012 over Inception-Resnet.

### 3.5. Comparison between the Optimal Model in this Paper and Traditional Machine Learning Methods under Different Characteristics

In this paper, 90% of the extracted deep feature samples and TIF feature samples were divided into training sets to train the machine learning algorithms, and 10% were divided into test sets for testing. The test results are shown in Figure 7. The SciKit-learn module in Python was used to implement random forest, SVM, and k-Means.

Combining Table 5 and Table 7 clearly shows that in different classification algorithms, the accuracy for TIFs was far lower than that of a single deep convolutional feature and even lower than that of a fused deep convolution feature. It can then be seen that TIFs represented a pathological image of the cervix. The classification ability was far weaker than that of a single deep convolutional feature, and the classification ability of a single deep convolutional feature was weaker than that of a fused deep convolutional feature.

## 4. Conclusions

In this paper, we noted that the accurate identification and classification of cervical cancer relies on the professional knowledge and analytical experience of pathologists. Missed detection and misdiagnoses often occur due to the high similarity in pathological cervical images, the large number of readings, the long reading time, and the insufficient experience levels of pathologists. Under this general background, we explored the underlying reasons for the low recognition accuracy of the existing computer-assisted methods of the recognition of pathological cervical tissue images and the insufficient recognition of the objects, and we solved the problem of existing models lacking feature extraction and expression capabilities and lacking deep learning with small samples. Their pathological classifications are not sufficiently detailed. Deep convolutional features were extracted by designing pre-trained ResNet50 V2, DenseNet121, VGG19, Inception V3, and Inception-ResNet deep network models, and LBP, HOG, and other algorithms were used to extract traditional image features of pathological cervical images. Decision fusion parameters, such as ET Weight, ANOVA F Weight, Chi2 weight, and other indicators, were then designed to qualitatively and quantitatively explore the differences between deep convolutional features and traditional image features in the representation of pathological cervical image samples. The new cervical cancer classification standard was explored to analyze the influence of the fusion of convolution features of different depths and the combination of different classifiers on the accuracy of pathological image classification. Finally, a pathological image classification algorithm for cervical cancer (AF-SENet) was formed by fusing convolutional features, and it is the first comprehensive identification algorithm for the various pathological stages of cervical cancer. Experiments proved that the optimal fusion of deep convolutional features proposed in this paper can better express pathological cervical images than traditional image features and shallow convolutional features. The proposed fusion subnet AF-SENet is effective against cervical cancer pathology. The four classification accuracy rates for the images reached 95.33%, which shows a higher clinical value than existing computer-aided algorithms. However, due to the rigor of clinical experiments and the difficulty of obtaining pathological data, the algorithm proposed in this paper has not been subjected to corresponding clinical double-blind verification experiments, and the overall recognition accuracy rate has room for further improvement.

## Figures and Tables

**Table 1:** Changes in the classification of precarcinoma of cervical squamous cell carcinoma.

Traditional	Version 3	Version 4
Mild atypical hyperplasia	CIN1	LSIL
Moderate atypical hyperplasia	CIN2	HSIL
Severe atypical hyperplasia	CIN3	HSIL
Carcinoma in situ	Carcinoma in situ	HSIL

**Table 2:** Model combinations and feature dimensions. (∪ represents features for serial fusion).

Numbering	Combined Content	Feature Length	Numbering	Combined Content	Feature Length
$C_{1}$	ResNet50 v2	2048	$C_{10}$	$C_{3}\cup C_{4}$	400
$C_{2}$	DenseNet121	1024	$C_{11}$	$C_{1}\cup C_{2}\cup C_{3}$	600
$C_{3}$	Inception v3	2048	$C_{12}$	$C_{1}\cup C_{2}\cup C_{4}$	600
$C_{4}$	Inception-ResNet	1536	$C_{13}$	$C_{2}\cup C_{3}\cup C_{4}$	600
$C_{5}$	$C_{1}\cup C_{2}$	400	$C_{14}$	$C_{1}\cup C_{2}\cup C_{3}\cup C_{4}$	800
$C_{6}$	$C_{1}\cup C_{3}$	400	TIF	—	1408
$C_{7}$	$C_{1}\cup C_{4}$	400	VGG19	—	512
$C_{8}$	$C_{2}\cup C_{3}$	400	LBP	—	256
$C_{9}$	$C_{2}\cup C_{4}$	400	HOG	—	1152

**Table 3:** Core parameters of each layer of the Analysis of Variance-F value-Spectral Embedding Net (AF-SENet model).

Layer Name	Number of Neurons	Excitation Function	Regularization
Inputs	Feature dimension	ReLu	L1
FC1	4096	ReLu	L1
BN1	—	—	—
FC2	4096	ReLu	L1
BN2	—	—	—
Classification	4	Softmax	—

**Table 4:** Software and hardware platforms.

Category	Name	Version
CPU	Intel Core I5 9600KF	—
GPU	NVIDIA GTX1070	—
Deep learning framework	Keras	V2.3.1

**Table 5:** Optimal test classification accuracy of pathological cervical tissue images (unit: %).

Feature	Normal	LSIL	HSIL	Cancer	Accuracy
ResNet50 v2	94.69	88.01	94.36	96.84	94.26
DenseNet121	95.45	88.79	92.58	96.42	94.28
Inception v3	93.07	87.37	93.60	95.94	92.96
Inception-ResNet	94.66	87.20	93.63	94.66	93.60
VGGNet19	83.67	53.21	73.34	82.91	78.37
TIF	75.78	47.12	46.12	66.20	68.31
$C_{5}$	96.00	90.89	94.57	97.13	95.33
$C_{6}$	94.45	88.32	93.01	95.87	93.75
$C_{7}$	95.11	87.81	92.63	95.61	93.87
$C_{8}$	94.64	88.77	93.02	95.95	93.91
$C_{9}$	95.02	88.16	92.97	95.47	93.91
$C_{10}$	95.39	89.36	93.72	96.45	94.57
$C_{11}$	94.82	89.91	93.36	96.38	94.28
$C_{12}$	95.08	88.32	93.17	95.50	94.01
$C_{13}$	95.16	89.09	93.41	95.92	94.28
$C_{14}$	95.18	89.06	93.53	96.08	94.33

**Table 6:** Model micro-AUC values.

Feature	Micro-AUC	Classifier	Micro-AUC
ResNet50 v2	0.9941	$C_{8}$	0.9891
DenseNet121	0.9949	$C_{9}$	0.9871
Inception v3	0.9891	$C_{10}$	0.9918
Inception-ResNet	0.9906	$C_{11}$	0.9898
VGGNet19	0.9506	$C_{12}$	0.9877
$C_{5}$	0.9959	$C_{13}$	0.9881
$C_{6}$	0.9866	$C_{14}$	0.9884
$C_{7}$	0.9847	TIF	0.8952

**Table 7:** Comparison of the classification accuracy of this article’s optimal deep feature fusion combination (ResNet50 v2-DenseNet121) and the machine learning methods of different features (four categories).

Classification Algorithm	$C_{5}$	TIF
AF-SENet	95.33%	68.31%
Random Forest	94.42%	63.54%
Support Vector Machines	94.88%	65.18%
k-Means	93.62%	86.77%

## Data Availability

Not applicable.
